# Supplementary material for: Noncontact Optical Measurement of Aqueous Humor Glucose Levels and Correlation with Serum Glucose Levels in Rabbit
Source: Biosensors (Basel). 2021 Oct 13;11(10):387. doi: 10.3390/bios11100387 (PMC8533889; doi:10.3390/bios11100387)
Supplement: Supplementary file 1 [file biosensors-11-00387-s001.zip › biosensors-1397330-supplementary.pdf]

Table S1. Aqueous glucose levels and serum glucose levels before (20 mins) and after (60 mins) the intravenous glucose challenge. Each rabbit had two observations.

.

Rabbit no.	Time (min)	Aqueous glucose (mg/dL)	Blood glucose (mg/dL)
1	20	169	191
	60	236	275
2	20	133	152
	60	171	204
3	20	127	152
	60	216	195
4	20	191	194
	60	253	217
